# Synergistic Anti-Cancer Activities of Curcumin Derivative CU17 Combined with Gemcitabine Against A549 Non-Small-Cell Lung Cancer Cells

**DOI:** 10.3390/pharmaceutics17020158

**Published:** 2025-01-24

**Authors:** Narissara Namwan, Gulsiri Senawong, Chanokbhorn Phaosiri, Pakit Kumboonma, La-or Somsakeesit, Arunta Samankul, Chadaporn Leerat, Thanaset Senawong

**Affiliations:** 1 Department of Biochemistry, Faculty of Science, Khon Kaen University, Khon Kaen 40002, Thailand; narissaranamwan@kkumail.com (N.N.); gulsiri@kku.ac.th (G.S.); s_arunta@kkumail.com (A.S.); l_chadapornee@kkumail.com (C.L.); 2 Department of Chemistry, Faculty of Science, Khon Kaen University, Khon Kaen 40002, Thailand; chapha@kku.ac.th; 3 Department of Applied Chemistry, Faculty of Science and Liberal Arts, Rajamangala University of Technology Isan, Nakhon Ratchasima 30000, Thailand; pakit.ku@rmuti.ac.th; 4 Department of Chemistry, Faculty of Engineering, Rajamangala University of Technology Isan, Khon Kaen 40000, Thailand; laor.so@rmuti.ac.th

**Keywords:** lung cancer, gemcitabine, curcumin derivative CU17, drug combination, cell cycle arrest, apoptosis, HDAC inhibitor, chemosensitizer

## Abstract

Recently, the curcumin derivative CU17 possessing HDAC inhibitory activity has been shown to synergistically enhance the anti-proliferative activity of Gem against lung cancer cells. Nevertheless, the mechanism(s) underlying the synergistic anti-cancer effect remains to be investigated. This study aimed to investigate the mechanisms that underpin the anti-cancer activity of the combined Gem and CU17 against NSCLC A549 cells both in vitro and in mouse xenograft models. CU17 was successfully synthesized and subsequently investigated for its combination effects with Gem on inductions of cell cycle arrest and apoptosis in A549 cells. The combination treatment substantially decreased cell survival through S phase prolongation and G2/M phase cell cycle arrest via up-regulating the expressions of p21 and p53 proteins. Additionally, CU17 potentiated the apoptotic effect of Gem in A549 cells by increasing the Bax/Bcl-2 ratio. The co-treatment resulted in an up-regulation of pERK1/2 and Ac-H3 expression. An in vivo study demonstrated that CU17 significantly improved the anti-cancer effect of Gem in nude mice utilizing A549 cell xenografts. The hematoxylin and eosin (H&E) staining results indicated that CU17 decreased the toxicity of Gem to the liver, kidneys, and spleen. Overall, CU17 enhanced the effectiveness of Gem while decreasing its toxicity. This compound shows promise as a chemosensitizer for NSCLC treatment with Gem.

## 1. Introduction

Globally, the mortality and morbidity rates associated with lung cancer are rather high. The predominant subtype of lung cancer is classified as non-small-cell lung cancer (NSCLC), accounting for 75–85% of all cases, while the remaining 15% is attributed to small-cell lung cancer (SCLC) [1]. The overall survival rates after five years for non-small-cell lung cancer patients are estimated to be approximately 15%. The delayed detection of the disease is one of the primary factors contributing to a decreased survival rate in lung cancer patients, resulting in the metastasization of the disease [2]. Chemotherapeutic strategies are the most frequently employed approach in treating malignancy of the lungs. Normally, gemcitabine (Gem) is considered a first-line chemotherapeutic option for lung cancer patients. Nevertheless, the beneficial effect of these approaches is frequently restricted by low drug absorption, which leads to an increase in metabolism in the first pass. Furthermore, these strategies have severe deleterious adverse effects and frequently develop drug resistance over time, limiting their clinical application [3]. Hence, it is essential to develop a treatment regimen that is both more effective and safer, combining a low dosage of Gem with additional adjuvant agents in order to reduce toxicity and prevent drug resistance [4].

Histone deacetylase (HDAC) inhibitor is one of the most advanced anti-neoplastic agents currently available, targeting epigenetic alterations. Histone acetyltransferases (HATs) and histone deacetylases (HDACs) particularly control the modification of histone acetylation in normal cells. However, carcinogenic cells frequently exhibit increased acetylation of oncogenes but decreased deacetylation of tumor suppressor genes. Thus, the inhibition of HDAC enzymes demonstrates a promising avenue for the treatment of malignant diseases [5]. Many HDAC inhibitors are undergoing clinical trials, and their effectiveness for single treatment in preclinical models demonstrates good tolerability and low toxicity in normal tissues. Nonetheless, the utilization of HDACi monotherapy has resulted in only moderate antitumor effects in clinical trials, particularly in the case of solid tumors [6]. The potential synergistic effects of HDAC inhibitors when combined with other chemotherapeutic agents have gained attention in order to improve their efficacy and avoid their adverse effects, such as toxicity and resistance [7]. Previously, several research investigations have demonstrated that combination treatments with HDAC inhibitors have improved the anti-cancer effects of traditional chemotherapeutic drugs by inhibiting a greater variety of cancer cell types. For instance, the proliferation of lung cancerous cells was substantially inhibited by the combination of SAHA and erlotinib via the apoptosis induction pathway [8]. Furthermore, the utilization of curcumin in combination with Gem, a chemotherapeutic agent employed for the treatment of developed lung carcinoma, in the management of non-small-cell lung cancer implies that curcumin, as a pharmacological adjuvant, may increase the sensitivity of Gem in lung cancer cells [4].

Curcumin derivative CU17 (CU17) is a newly synthesized amino derivative of curcumin. Based on our prior investigations, it was observed that CU17 inhibited the activity of the HDAC enzyme derived from HeLa nuclear extract [9]. This inhibition was observed to be dose-dependent, with an IC_50_ value of 0.41 µM. In addition, the increased acetylation of histone H3 (Ac-H3) in NSCLC cells is a consequence of CU17’s potent inhibitory effect on HDAC. In addition, CU17 has been reported to have a cytotoxic effect on NSCLC cells, exhibiting anti-cancer potential through cell cycle arrest in the G2/M phase and apoptosis cell death pathways. In addition, CU17 could potentiate the anti-proliferative activity of Gem to suppress the proliferation of NSCLC cells. The sub-toxic doses of Gem (the dose causing growth inhibition of approximately 20% and 30%) combined with different concentrations of CU17 demonstrated a synergistic impact on lung cancer cells at 48 h exposure. However, the mechanism(s) underlying the synergistic drug interactions between CU17 and Gem against lung cancer cells has not been well explored. Therefore, the aim of this study was to investigate the anti-cancer mechanism(s) of the combination treatments of CU17 and Gem against human lung cancer A549 cells both in vitro and in vivo.

## 2. Materials and Methods

### 2.1. Materials

Turmeric rhizome powder was purchased from a natural pharmacy in Khon Kaen province, Thailand. Fetal bovine serum (FBS) was bought from Cytiva (Kremplstrasse, Pasching, Austria), while RPMI-1640 medium, trypsin-EDTA, and penicillin/streptomycin were procured from Thermo Fisher Scientific Inc. (Grand Island, NY, USA). Annexin V-FITC was purchased from Biolegend (San Diego, CA, USA), and 3-(4,5-dimethylthiazol-2-yl)-2,5-diphenyltetrazolium bromide (MTT) was acquired from Invitrogen (Eugene, OR, USA). Olive oil, propidium iodide (PI), and gemcitabine hydrochloride (Gem) were all obtained from Sigma-Aldrich Corporation (St. Louis, MO, USA). In addition, Cell Signaling (Beverly, MA, USA) provided the primary antibodies (anti-p53, anti-Bcl-2, anti-Bax, anti-Ac-H3, anti-p21, anti-pERK1/2, anti-Total ERK1/2, and anti-β-actin) and the secondary antibodies (anti-mouse IgG and anti-rabbit IgG conjugated to horseradish peroxidase).

### 2.2. Cell Lines and Culture Conditions

The human lung adenocarcinoma A549 cell line was generously provided by Prof. Dr. Arunporn Ittharat (Department of Applied Thai Traditional Medicine, Faculty of Medicine, Thammasat University, Bangkok, Thailand). RPMI-1640 medium was used to culture the cells. Furthermore, 10% FBS, 100 U/mL penicillin, and 100 μg/mL streptomycin were added to the medium to promote the cell culture. The cells were also cultured at 37 °C in a humidified condition with 5% CO_2_.

### 2.3. The Extraction and Isolation of Curcumin

Curcumin was produced for the synthesis of CU17, which was obtained by extracting and isolating turmeric root, as previously reported [10]. Briefly, the extraction of powdered turmeric was performed three times with dichloromethane (CH_2_Cl_2_) (1000 mL/time). Subsequently, dichloromethane extract was obtained by condensing the extract in a rotary evaporator at low pressure. Silica gel column chromatography was employed to separate the dichloromethane extract. Then, the extract was eluted with hexane, ethyl acetate (EtOAc), and methanol (MeOH). The elution solutions were collected and divided into four fractions, including DT1, DT2, DT3, and DT4. The DT2 fraction was subjected to flash silica gel column chromatography and a hexane-EtOAc gradient elution (from 10:0 to 5:5) in order to produce three sub-fractions (DT2-1 to DT2-3). Afterwards, DT2-1 and DT2-2 were identified via thin-layer chromatography employing a hexane-EtOAC (9:1) mobile phase for purification. The resulting products were demethoxycurcumin and dihydrocurcumin, which had a solid yellow color. The separation of DT2-3 was performed using silica gel column chromatography and CH_2_Cl_2_-MeOH (9:1) mobile phase to obtain curcumin as a solid orange color. The NMR data of all products were similar to those reported by Venkateswarlu [11].

### 2.4. Curcumin Derivative CU17 Synthesis

The synthesis of CU17 was performed according to the previous procedure [10]. Briefly, curcumin was dissolved in ethanol. Thereafter, 2-aminothiophenol was added to the curcumin solution, which was then mixed at room temperature. After refluxing for 6 h, a combined solution was filtered, washed, dried, and evaporated. Finally, the reaction mixture was purified by column chromatography via elution with 5% MeOH in CH_2_Cl_2_ to yield (4Z,6E)-5-Hydroxy-1,7-bis(4-hydroxy-3-methoxyphenyl)-1-((2-mercaptophenyl)amino)hepta-4,6-dien-3-one (CU17) as a solid yellow. The NMR and HPLC results corresponded with our previously published data [9,10]. NMR, IR, and mass spectra are provided in the Supplementary Materials.

### 2.5. Cell Viability Assay

The effect of CU17 on cell proliferation was evaluated by MTT assay as described previously [9]. A cellular density of 8 × 10^3^ cells/well was seeded onto a 96-well plate and incubated at 37 °C for 24 h. Thereafter, the cells were exposed to different concentrations of CU17 for durations of 24, 48, and 72 h. The treated cells were then incubated with a fresh medium supplemented with MTT for three hours at 37 °C. After the removal of the medium, DMSO was used to dissolve the formazan crystals. The absorbance of the formazan solution was measured at 570 nm using a microplate reader (iMark™ Microplate Absorbance Reader, Bio-Rad, Hercules, CA, USA), with 655 nm serving as the reference wavelength. The percentages of cell viability were calculated by utilizing the equation below.

$$\mathrm{Cell}\;\mathrm{viability}\;(\%)=\;\frac{A_{570\mathrm{treatment}}-{O.D.}_{655\mathrm{treatment}}}{A_{570\mathrm{solvent}\;\mathrm{control}}-{O.D.}_{655\mathrm{solvent}\;\mathrm{control}}}\times 100$$

where A and O.D. are the absorbance and optical density, respectively.

### 2.6. Cell Cycle Analysis

The effects of the co-treatment between Gem and CU17 on cell cycle progression were evaluated using the standard flow cytometric technique. A549 cells were seeded onto a 5.5 cm dish plate at a density of 1 × 10^6^ cells/dish and subsequently subjected to treatment with Gem and CU17 alone or in combination at a synergistic condition (48 h exposure), as previously mentioned [9]. Briefly, A549 cells were treated with solvent (0.5% ethanol + 0.5% DMSO), Gem (0.68 and 1.30 µM), CU17 (0.75 and 0.91 µM), Gem (0.68 µM) + CU17 (0.91 µM), and Gem (1.30 µM) + CU17 (0.68 µM) for 48 h. After that, the cells were harvested using trypsin at 37 °C and immediately fixed in a 75% ice-cold ethanol solution on ice for 1 h. The cells were then rinsed and resuspended in RNase A solution at 37 °C for 30 min prior to being stained with PI at room temperature for 45 min. Finally, the stained cells were assessed using the BD FACSCanto II flow cytometer (Becton Dickinson, San Jose, CA, USA) to identify cell cycle progression.

### 2.7. Evaluation of Apoptosis Induction

The fluorescein isothiocyanate (FITC)-conjugated Annexin V and propidium iodide (PI) double-staining assay was utilized to determine apoptosis induction. The A549 cells were grown on a dish plate and then exposed to treatment for a period of 48 h with Gem and CU17 alone or in combination at a synergistic condition (48 h exposure), as previously published [9]. The treated cells were then collected using trypsin at 37 °C and stained with Annexin V-FITC and PI. The apoptotic cells were detected using the BD FACSCanto II flow cytometer (Becton Dickinson, San Jose, CA, USA).

### 2.8. Western Blot Analysis

A549 cells were seeded at a concentration of 1 × 10^6^ cells/dish and cultured for 24 h. Cells were then treated with Gem and CU17 alone or in combination for 48 h at a synergistic condition, as previously published [9]. Thereafter, cells were initially collected with a scraper and lysed in a lysis buffer containing protease inhibitors, and the lysates were maintained on ice. A Bio-Rad protein assay (Bio-Rad, Hercules, CA, USA) was then used to assess the concentration of the protein. Equal amounts (30–50 μg) of total protein from each treatment were resolved by SDS-PAGE and transferred to the polyvinylidene difluoride (PVDF) membrane. The membranes were blocked using a 3% nonfat milk solution for 1 h at room temperature. Afterward, PVDF membrane was incubated overnight at 4 °C with the following primary antibodies: anti-p53 (#2524, diluted 1:1000), anti-Bcl-2 (#4223, diluted 1:1000), anti-Bax (#2772, diluted 1:1000), anti-Ac-H3 (#9649, diluted 1:1000), anti-p21 (#2946, diluted 1:1000), anti-pERK1/2 (#9102, diluted 1:1000), and anti-total ERK1/2 (#9107, diluted 1:2000) (Cell Signaling, Beverly, MA, USA). The membranes were rinsed in 1X PBST for 2 min two times and then incubated with HRP-linked goat anti-mouse (#7076, diluted 1:2000) or anti-rabbit (#7074, diluted 1:2000) secondary antibodies at room temperature for 2 h following 1X PBST and PBS washes (2 × 2 min each), respectively. Ultimately, the blot was observed through the utilization of enhanced chemiluminescence reagents (Bio-Rad, Hercules, CA, USA), and X-ray films were employed to analyze the immunoreactive bands. The measurement of relative intensity was performed using the ImageJ program, while β-actin was employed as a loading control for Western blotting to normalize the levels of protein; total ERK1/2 was employed as a loading control for pERK1/2 protein expression.

### 2.9. In Vivo Anti-Tumor Study

Female nude mice (BALB/CAJcl-Nu/Nu, 6–7 weeks old) were procured from Nomura Siam International, Bangkok, Thailand. The handling and care of mice were controlled in the Institutional Animal Care and Use Committee of Khon Kaen University and allowed by the Animal Research Committee of the institution. Human lung cancer A549 cells (6 × 10^6^ cells in 0.1 mL of serum-free medium mixed with Matrigel) were subcutaneously implanted in the right axillary flank of mice. Mice were randomly separated into six groups (n = 5) once tumor formation had reached 100 mm^3^. Firstly, group 1 was the vehicle control group, receiving 5% DMSO in olive oil. Group 2 was the Gem treatment group, receiving 50 mg/kg of Gem, while group 3 was the CU17-15 group, receiving CU17 15 mg/kg. Group 4 was the CU17-30 group, receiving CU17 30 mg/kg, whereas group 5 was the Gem+CU17-15 group, receiving 50 mg/kg of Gem and 15 mg/kg of CU17. Lastly, group 6 was the Gem+CU17-30 group, receiving 50 mg/kg of Gem and 30 mg/kg of CU17. The mice were intraperitoneally administered Gem and CU17 alone or in combination every three days for twenty-one days. Tumor size and body weight were monitored during the study. The dimensions of the tumors were assessed utilizing calipers in every experiment, while the volume of tumors was calculated employing the subsequent formula: Volume of tumor (mm^3^) = (a × b^2^)/2, where a indicates the length and b indicates the width of the tumor in mm. At the end of the experiment, the mice were sacrificed, and the xenograft tumors, kidneys, liver, and spleen were harvested and weighed. The relative tumor volume (RTV), tumor growth inhibition ratio (TGI, %), and body weight change (BWC) were determined according to the methodology outlined in the prior publication [12].

### 2.10. Histological Examination

After 48 h of fixation in 10% formalin, the tumors, liver, kidneys, and spleen were embedded in paraffin, sliced every 4 µm by microtome, and subsequently transferred to glass slides. After dewaxing in xylene, the tissue was dehydrated using alcohol in reducing concentrations (99, 95, and 70%) prior to rinsing with distilled water. Hematoxylin and eosin (H&E) were utilized to stain the rehydrated tissue sections. Then, inverted fluorescence microscopy (Olympus BX60 Fluorescence Microscope) (Olympus Corporation, Tokyo, Japan) was used to capture the representative areas at 400 magnifications.

### 2.11. Statistical Analysis

All experiments were performed independently in triplicate. The results are shown as the mean ± standard deviation (SD) or standard error of the mean (SEM). To compare means of continuous variables between groups, datasets were analyzed using the statistical program IBM SPSS version 26.0 for Windows (SPSS Corporation, Chicago, IL, USA). To compare significant differences between the solvent control and treatment groups, one-way analysis of variance and Dunnett’s t-test were used. Statistical significance was considered at *p* < 0.05.

## 3. Results

### 3.1. Synthesis of CU17

**Figure 1:**
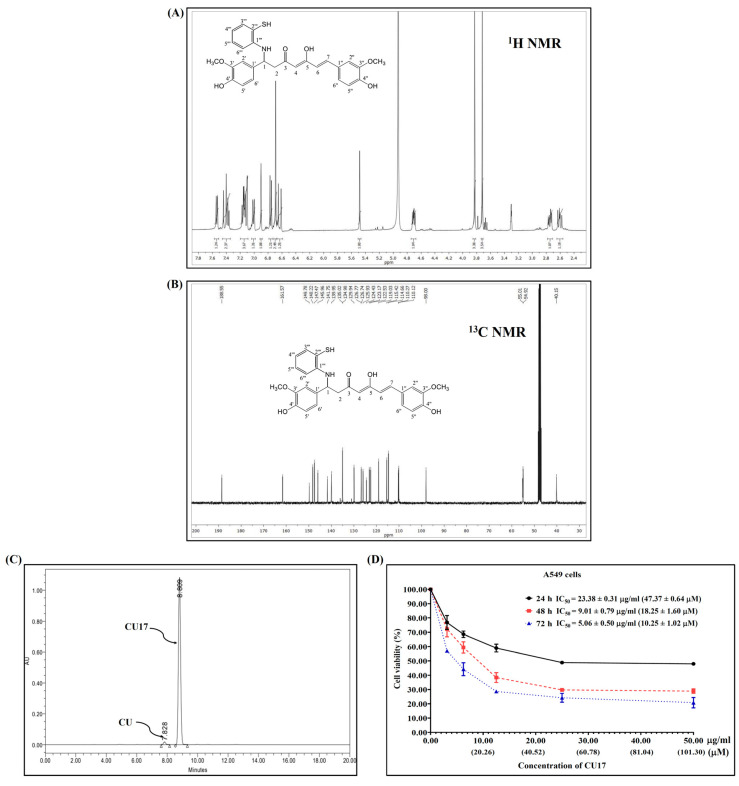
The ^1^H and ^13^C NMR spectra (**A**,**B**). HPLC chromatogram (**C**) of CU17 and its anti-proliferative effect against A549 cells (**D**). A549 cells were exposed to CU17 at varying concentrations for 24, 48, and 72 h. Data are expressed as mean ± SEM of three independent experiments conducted in triplicate.

The synthesis and characterization of CU17 were carried out as previously reported [10], and the ^1^H and ^13^C NMR were used to confirm the chemical structure of CU17 (Figure 1A,B). RP-HPLC was utilized to determine the quantity of CU17 via UV–vis detection at 425 nm. Additionally, the RP-HPLC results indicated that CU17 was identified as the majority of the purified products, with a tiny amount of curcumin (Figure 1C). The retention times for CU and CU17 were 4.9 and 5.7 min, respectively, and their respective area percentages were 1.8 and 98.2%.

### 3.2. Anti-Proliferative Effect of CU17 in Human Lung Cancer A549 Cells

The inhibitory effects of CU17 on the proliferation of A549 cells were determined using the MTT assay. The inhibition of cell proliferation was observed at all concentrations of the drug tested at 24, 48, and 72 h. The proliferation of A549 cells was inhibited under CU17 post-treatment in a dose-dependent manner (Figure 1D). The half maximal inhibitory concentration (IC_50_) values for CU17 in A549 cells at 24, 48, and 72 h were 23.38 ± 0.31 μg/mL (47.37 ± 0.64 μM), 9.01 ± 0.79 μg/mL (18.25 ± 1.60 μM), and 5.06 ± 0.50 μg/mL (10.25 ± 1.20 μM), respectively. The IC_50_ values of CU17 against the lung adenocarcinoma cell line (A549) were consistent with the values reported in a previous study [9]. The new lot of synthesized CU17 used in this study exhibited identical chemical structure and anti-cancer effects to that of the previous lot. Therefore, we further explored the underlying anti-cancer mechanisms of synthesized CU17 in combination treatments with Gem against A549 cells.

### 3.3. CU17 Enhances Gem-Induced Cell Cycle Arrest in Human Lung Cancer A549 Cells

**Figure 2:**
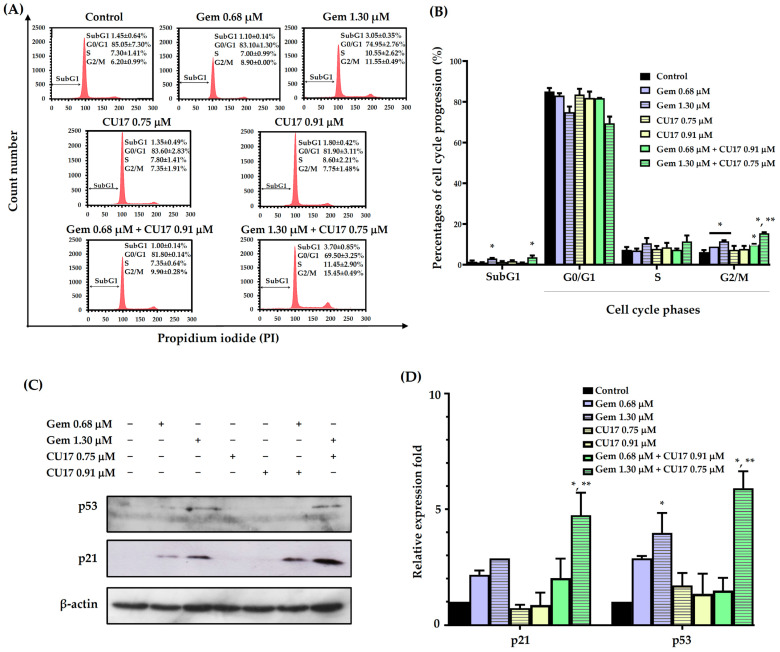
The effects of single and combination drug treatments of Gem and CU17 on cell cycle progression in lung cancer A549 cells. Cells were subjected to single-drug treatment and co-treatment between Gem (0.68 and 1.30 µM) and CU17 (0.75 and 0.91 µM) for a duration of 48 h. The cell cycle distribution was assessed using flow cytometry utilizing PI labeling. The representative histograms displayed the distribution of A549 cells based on their DNA content after treatments (**A**). A control was established using a solvent treatment consisting of 0.5% ethanol and 0.5% DMSO (*v*/*v*). Percentages of cell distribution in each phase of the cell cycle were displayed in bar graphs (**B**). Protein expression levels of p21 and p53 (**C**) and their relative expression fold (**D**) were displayed, where the β-actin was employed as a loading control. All results were presented as mean ± SD (n = 3, * *p* < 0.05 compared to the control, and ** *p* < 0.05 compared to the single treatment).

In order to examine the potential synergistic effects of Gem and CU17 in suppressing cell proliferation by inducing cell cycle arrest, we used the propidium iodide (PI) staining method to assess the cell cycle distribution in A549 cells. The progression of the A549 cell cycle was evaluated in relation to the dosage and duration of co-treatment exposure, using a synergistic condition as previously published [9]. The results revealed that the co-administration of 0.68 µM Gem and 0.91 µM CU17 resulted in a greater proportion of cells arresting at the G2/M phase (9.90 ± 0.28%) when compared with CU17 single-drug treatment (7.75 ± 1.48%). Significantly, a higher proportion of cells halted at the S (11.45 ± 2.90%) and G2/M phases (15.45 ± 0.49%) was observed in the combination treatment of Gem at 1.30 µM and CU17 at 0.75 µM when compared with CU17 single-drug treatment (S = 7.80 ± 1.41 and G2/M = 7.35 ± 1.91%). Particularly, the co-administration between Gem at 1.30 µM and CU17 at 0.75 µM caused an increased apoptotic cell population (3.70 ± 0.85%) in comparison to CU17 single-drug treatment (1.35 ± 0.49%) (Figure 2A,B).

We further investigated the expressions of cell cycle-associated proteins, including p53 and p21. The expression of p21 and p53 proteins was not significantly altered by the combined administration of 0.68 μM Gem and 0.91 μM CU17 when compared with those of the single and vehicle treatments (Figure 2C,D). However, the combination treatment between 1.30 µM Gem and 0.75 µM CU17 caused the up-regulation of p53 and p21 compared to the effect of every single agent. These results suggested that the combination of Gem and CU17 triggered cell cycle arrest at the S and G2/M phases in lung cancer A549 cells via up-regulating p53 and p21 protein expression.

### 3.4. CU17 Potentiated the Apoptosis Effect of Gem in Human Lung Cancer A549 Cells

**Figure 3:**
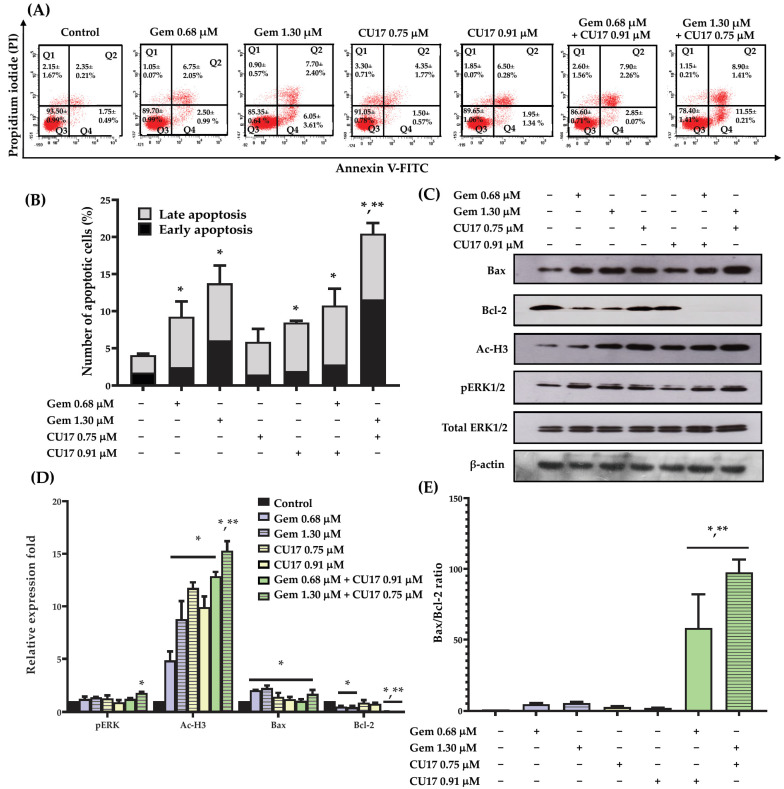
The effects of single and combination drug treatments of Gem and CU17 on apoptosis induction in lung cancer A549 cells. Cells were exposed to CU17 and/or Gem for 48 h. The presence of apoptotic cells was identified using Annexin V-FITC/PI test. Dot plots depicting the representative Annexin V-FITC/PI data from three consistent trials were shown (**A**). Percentage of apoptotic cells after the indicated treatment was presented in bar graph (**B**). The protein expression levels of pERK1/2, Ac-H3, Bax, and Bcl-2 in A549 cells after various treatments were assessed using Western blot analysis. The relative optical densities of these proteins were quantified using ImageJ (**C**,**D**). The expression ratio of Bax/Bcl-2 was reported (**E**), where the β-actin was employed as a loading control for protein expression and total ERK1/2 was employed as a loading control for pERK1/2 protein expression. All results were expressed as mean ± SD (n = 3, * *p* < 0.05 compared with control and ** *p* < 0.05 compared with single treatment).

To examine the potential synergistic effects of Gem and CU17 in suppressing cell proliferation by inducing cellular apoptosis, we used the Annexin V-FITC/PI assay and flow cytometry to assess apoptosis induction in A549 cells. Based on the results from the Annexin V-FITC/PI assay, Annexin V-FITC-positive cells with PI-negativity are classified as early apoptotic cells, whereas Annexin V-FITC-positive cells with PI-positivity are identified as late apoptotic cells. A549 cells treated with CU17 (0.75 and 0.91 µM) exhibited an increase in apoptotic cell populations (both early and late apoptosis) of 5.85 ± 0.85 to 8.45 ± 0.75%, respectively (Figure 3A,B). The combination treatment with CU17 (0.91 µM) and Gem (0.68 µM) caused no significant increase in apoptosis induction against A549 cells (10.75 ± 1.55%) when compared to Gem treatment alone (9.25 ± 0.75%). However, the treatment combining Gem (1.30 μM) and CU17 (0.75 μM) caused a significant increase of apoptosis in A549 cells (20.45 ± 0.85%) compared to Gem treatment alone (13.75 ± 0.85%). This discovery illustrated that the potency of CU17 to improve the anticancer activity of Gem against A549 cells may be attributed to synergistic apoptosis induction.

In order to acquire an extensive understanding of the mechanisms that contribute to the induction of apoptosis in A549 cells of the combined CU17 and Gem, Western blot analysis was employed to investigate the alteration of associated apoptotic protein expression in A549 cells. The Western blot results demonstrated that not all treatments altered the expression level of the Bax protein when compared to the control treatment. However, the expression level of the Bcl-2 protein was significantly reduced in the combination treatment compared to the single-drug and solvent control treatments (Figure 3C,D). Interestingly, the combination treatment exhibited a much greater Bax/Bcl-2 expression ratio than either of them alone (Figure 3E). Thus, CU17 synergized the chemotherapeutic effect of Gem-induced apoptosis via regulating Bax/Bcl-2 signaling in lung cancer A549 cells. Furthermore, the administration of Gem (1.30 μM) and CU17 (0.75 μM) resulted in the substantial up-regulation of pERK1/2 and Ac-H3 protein expression in A549 cells compared to the single drug and solvent control treatments.

### 3.5. CU17 and Gem Combination Inhibited A549 Xenograft Tumor Growth In Vivo

**Figure 4:**
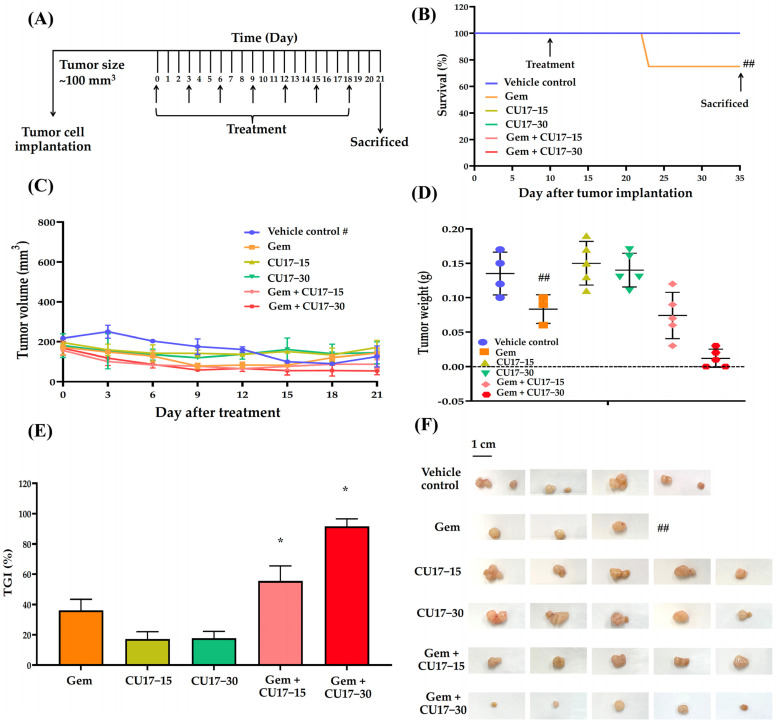
CU17 enhanced the antitumor activity of Gem in a mouse xenograft model. The experimental design included the administration of Gem (50 mg/kg) and CU17 (15 and 30 mg/kg) alone, as well as their combination (**A**). After 21 days of therapy, the survival rate of all xenograft mice was measured. The arrows indicate the starting point and termination of treatment (**B**). Representatives of tumor inhibition (**C**), tumor volume (**D**), and tumor weight (**E**) after treatment with the indicated drugs were demonstrated. The tumor xenografts were surgically eliminated at the end of the observing period (**F**). * *p* < 0.05, compared with vehicle control (vehicle control, n = 4; Gem, n = 3; CU17-15, CU17-30, Gem+CU17-5, and Gem+CU17-30, n = 5). # presents the tumor volumes of the vehicle control group measured at the primary site (metastasis of the vehicle-treated tumors was observed after day 12). ## displays the death of mice in the Gem group during treatment, resulting in a total of three mice remaining at the end.

Considering our in vitro results, we anticipate that CU17 may enhance the therapeutic activity of Gem against the A549 NSCLC xenograft mouse model. Tumor growth was measured using a digital vernier caliper after a subcutaneous injection of A549 cells into the mice with a weight between 23 and 25 g. In this study, one mouse died from a hepatic abscess while the tumors were growing, leaving 28 mice in total. The mice were randomized into six treatment groups after the tumor size reached 100 mm^3^: the vehicle control (n = 4), Gem (n = 4), CU17-15 (n = 5), CU17-30 (n = 5), Gem+CU17-15 (n = 5), and Gem+CU17-30 (n = 5). For 21 days of treatment, mice were administered intraperitoneal (i.p) injections containing 5% DMSO in olive oil (serving as the vehicle control), Gem, CU17-15, or CU17-30 alone or in combination every 3 days (Figure 4A). During treatment, only one mouse died from the Gem group but none of the mice in the other groups died (Figure 4B). The tumors were removed from the sacrificed mice and photographed once the treatment was finished (Figure 4F). As anticipated, mice receiving the combination therapy exhibited smaller tumor volumes than those receiving monotherapies. Nevertheless, mice in the vehicle group revealed a decrease in tumor volume at the primary site after day 12 (Figure 4C). Over a period of 21 days, the co-administration of Gem and CU17 (15 and 30 mg/kg) caused a substantial reduction in tumor weight when compared to vehicle treatment (Figure 4D). Furthermore, the co-administration of Gem and CU17 at doses of 15 or 30 mg/kg dramatically inhibited tumor growth by 55.56 ± 17.35 and 91.67 ± 8.34%, respectively (Figure 4E). Overall, Gem and CU17 act synergistically to inhibit tumor growth in nude mice.

### 3.6. Toxicity Evaluation of CU17 in Combination with Gem In Vivo

The evaluation of the systemic negative effects of pharmaceuticals on mice during treatment included monitoring changes in body weight, organ weight, and the histology of the liver, kidneys, and spleen. The starting and ending body weights exhibited no statistically significant differences between the vehicle control and all the treatment groups. The single and combination treatments did not result in any significant changes in the weights of the liver and kidneys when compared to the vehicle control group. Additionally, the spleen weights of the mice treated with CU17 alone were not decreased. In contrast, Gem alone and its combination treatments resulted in significant increases in spleen weights when compared to the vehicle control group (Table 1).

**Figure 5:**
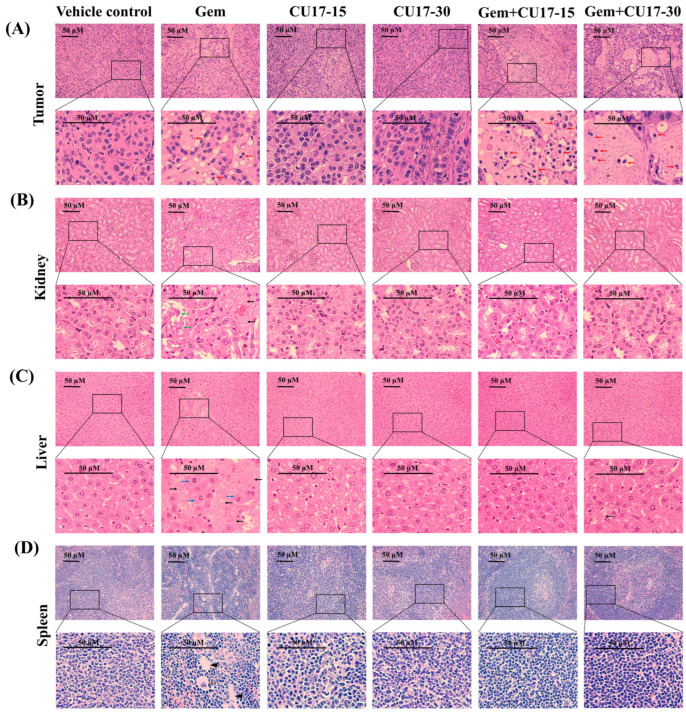
Assessment of biotoxicity in nude mouse xenograft. The tissues, including the tumor (**A**), kidneys (**B**), liver (**C**), and spleen (**D**), were subjected to hematoxylin and eosin staining. The pathogenic abnormalities were seen using inverted fluorescence microscopy at a magnification of ×400, with a scale bar of 50 µm. Red arrow represents apoptotic cells, black arrow represents necrosis, green arrow represents disrupted basal membrane, blue arrow represents hypertrophy, orange arrow represents megakaryocytes, head arrow represents focal fibrosis, and star represents cell death.

In addition, we analyzed the cellular morphology as well as tissue structure of the tumor tissue and organs considered the primary targets of drug toxicity, including the liver, kidneys, and spleen. Histological examination of tumor tissues obtained from the groups treated with Gem (50 mg/kg) and CU17 (15 and 30 mg/kg) revealed a substantial decrease in the number of proliferating cells and a high-power image depicting the morphological characteristics associated with apoptosis (red arrow) in comparison to the control and single-treatment groups (Figure 5A). In addition, the co-treatment caused a greater number of cell deaths than Gem monotherapy, which led to the development of a uniform pink region (star). Further examination of kidney tissue following intraperitoneal Gem injection revealed necrosis (black arrows) and a disrupted basal membrane (green arrow) (Figure 5B). Additionally, necrosis and hypertrophy (blue arrow) were observed in the hepatocyte architecture in response to Gem treatment alone (Figure 5C). Tissue sections of the spleen from mice receiving Gem monotherapy revealed extramedullary hematopoiesis, showing megakaryocytes (orange arrow) with focal fibrosis (head arrows) (Figure 5D). In combination treatments with CU17, cells with necrosis were essentially absent and cells with a regular cell structure were more observed. Overall, CU17 synergistically enhanced Gem activity to inhibit tumor growth. Moreover, CU17 reduced the toxicity of Gem and protected the functions of the main organs.

## 4. Discussion

NSCLC is a common clinical type of lung cancer, with a high incidence rate [13]. Chemotherapy is the most common method of treating NSCLC in clinical practice, in which Gem is used as one of the main chemotherapeutic medications in the treatment strategy [14]. Nevertheless, Gem-related resistance is frequently identified as an important limitation of lung cancer treatment [15]; therefore, the therapeutic efficacy of Gem requires further enhancement. Several studies demonstrated that HDAC inhibitors exhibited the potential to enhance the prevention and treatment of cancer and also offered outstanding safety levels [16,17,18,19,20,21,22]. Moreover, the synergistic effects of HDAC inhibitors with Gem have been studied to improve effectiveness and reduce toxicity and resistance [23,24,25,26,27]. A previous study revealed that the anticancer effects of Gem combined with the HDAC inhibitor CUDC-101 were more effective against pancreatic cancer than the single treatment [3]. Additionally, we reported that the combination of CU17 and Gem exhibited synergistic anticancer effects in A549 cells [9]; however, the molecular mechanisms underlying the synergistic activity of CU17 and Gem against human lung A549 carcinoma cells are yet unclear. This current study assessed the anticancer mechanisms of CU17 in combination with Gem against human lung cancer A549 cells in vitro and also evaluated the combination effect of CU17 with Gem in a mouse xenograft model. The synthesis of CU17 employed in this study was carried out using previously published methods [10], and Figure 1A,B revealed data proving a successful synthesis and purity. CU17 inhibited the proliferation of A549 cells with IC_50_ values of 23.38 µg/mL (47.37 µM), 9.01 µg/mL (18.25 µM), and 5.06 µg/mL (10.25 µM) for exposure times of 24, 48, and 72 h, respectively (Figure 1C).

The biological effects of CU17 coupled with Gem on the regulation of the cell cycle progression and induction of apoptosis were further examined. Malignant transformation is strongly linked to cell cycle disruption and cell cycle arrest and may decrease cancer cell growth and lead to cell death; hence, the regulation of cell cycles may be linked to cancer prevention [28,29]. In this present study, we found that Gem (0.68 µM) combined with CU17 (0.91 µM) promoted greater A549 cell accumulation in the G2/M phase than each of the drugs alone. Furthermore, combining Gem (1.30 µM) and CU17 (0.75 µM) was shown to halt cell cycle progression at the S and G2/M phases (Figure 2A,B). p21 is a potent cyclin-dependent kinase inhibitor (CKI) that directly binds to cyclin-CDK2, cyclin-CDK1, and cyclin-CDK4/6 complexes to block their activities [30]. The down-regulation of p21 is linked to tumor invasion, metastasis, differentiation, and proliferation [30]. Drug-resistant phenotypes after cancer treatment are also associated with abnormal p21 protein expression [30]. Therefore, targeting p21 may be an effective treatment for malignant growth. The tumor suppressor transcription factor p53 has been reported to detect DNA damage and determine apoptosis or cell survival. Recently, p53 was shown to stimulate p21 expression, which caused cell cycle arrest [31]. In this study, the combined treatment of Gem (1.30 µM) and CU17 (0.75 µM) significantly increased the expression of p53 and p21 proteins compared to single-drug treatment (Figure 2C,D). Hence, CU17 increased the sensitivity of A549 cells to Gem by modulating p21 expression via a p53-dependent pathway.

Apoptosis pathways are crucial for the prevention and treatment of cancer, as well as for assessing the efficacy of anticancer drug actions [32]. Apoptosis causes cell shrinkage, chromatin condensation, and plasma membrane blebbing [33]. There are two main mechanisms that regulate apoptosis: death receptor-induced extrinsic and mitochondria-driven intrinsic pathways [34]. The intrinsic apoptotic pathway is initiated by the Bcl-2 family, which includes pro-apoptotic (Bax, Bad, and Bak) and anti-apoptotic (Bcl-2, Bcl-xL, and Bcl-B) proteins. Tumor cells attain apoptosis resistance by down-regulating Bax and up-regulating Bcl-2. Many types of malignancies missed p53, which controlled Bax and Bcl-2 [35,36]. Our findings revealed that co-treatment with CU17 and Gem significantly caused increased apoptosis in A549 cells compared to single and control treatments (Figure 3A,B). Furthermore, CU17 in combination with Gem did not change the level of the pro-apoptotic Bax protein but significantly caused a decreased level of the anti-apoptotic Bcl-2 protein (Figure 3C,D). Moreover, statistical analysis demonstrated that the co-administration of CU17 and Gem resulted in a substantially increased ratio of pro-apoptotic Bax protein to anti-apoptotic Bcl-2 protein when compared to the single drug treatments (Figure 3E). Additionally, p21 prevents cell invasion and promotes apoptosis by up-regulating Bax and Bak and down-regulating Bcl-2 and Bcl-XL [37,38]. A previous study demonstrated that CU treatment caused increased pro-apoptotic activity in PANC-1 cells, resulting in their being sensitive to Gem [39]. In addition, Gem and SFN-mediated HDAC inhibition caused increased Bax expression and decreased Bcl-2 expression in intrahepatic cholangiocarcinoma HuCCT-1 and HuH28 cells [40]. Our findings suggest that the combination of Gem and CU17 led to increased cellular apoptosis, resulting in improved sensitivity of Gem in A549 cells through the activation of Bax/Bcl-2-dependent intrinsic apoptosis.

One of the most important intracellular signaling pathways for tumor metastasis, cell proliferation, differentiation, apoptosis, and angiogenesis is the ERK pathway. ERK hyperactivation is a characteristic of numerous malignancies, and its deregulated activity stimulates cell proliferation [41]. However, previous studies demonstrated that the upregulation of p53 and the modulation of Bcl-2 family proteins in a MEK-dependent manner were correlated with apoptosis induced by a variety of DNA-damaging agents [42,43,44]. This suggests that ERK activation stimulates p53 transactivation. Furthermore, ERK activation links p53 phosphorylation at Ser15 to p53 up-regulation/accumulation. This process stabilizes p53 by preventing it from adhering to Mdm2, a p53-ubiquitin ligase. The findings of this study demonstrated that CU17 (0.91 µM) and Gem (0.68 µM) combination treatment did not significantly alter ERK 1/2 phosphorylation in A549 cells. In contrast, the co-treatment with CU17 at 0.75 µM and Gem at 1.30 µM significantly raised ERK 1/2 phosphorylation in A549 cells (Figure 3C,D). These findings suggest that the synergistic effect of CU17 on Gem-treated A549 cells may have been achieved through ERK signaling pathway activation, leading to p53 phosphorylation and Bcl-2 downregulation. HDAC inhibitors have been shown to cause decreased tumor cell proliferation, differentiation, and death but have minimal impact on normal tissue [45]. HDAC activation may cause molecularly targeted treatment and chemotherapeutic resistance. Thus, combining HDAC inhibitors with current anticancer drugs to reverse resistance or enhance effectiveness is garnering interest. HDAC inhibitors caused the increased p21 and DNA damage in NSCLC cells, enhancing the effects of carboplatin [46]. Our previous study demonstrated that CU17 inhibited HeLa nuclear extract HDAC enzymes and caused hyperacetylated histone H3 (Ac-H3) in A549 cells, indicating the HDAC inhibitory activity of CU17 [9]. Earlier DRUGSURV searches found that lung cancer patients with increased HDAC1, HDAC2, and HDAC6 had a poor prognosis [47]. In this study, treating A549 cells with combined CU17 at 0.75 μM and Gem at 1.30 μM led to a significant increase of histone H3 hyperacetylation when compared to that of the single-drug treatments (Figure 3C,D). Newbold et al. reported that HDAC inhibitors also caused the up-regulation of p21 to stop the cell cycle [48]. Thus, Ac-H3 up-regulation may contribute to the increased promotion of apoptosis and inhibition of cell cycle progression by Gem and CU17 combination treatment.

Finally, A549 cells were injected subcutaneously into nude mice in order to prove the effectiveness of CU17 in both single and combination treatments in vivo. After 21 days of treatment, the combined CU17 and Gem treatment showed a significant increase in tumor growth inhibition (%), reducing tumor weight (Figure 4C–F). However, the co-treatment showed no significant effect on body weight when compared to the group that received a single treatment (Table 1). The unexpected decline of tumor volume in the vehicle group may be attributed to the widespread lung cancer metastases. According to Otani et al., histological and CT imaging revealed lung metastases in mice implanted with A549 cells but not FT821 or PC-9 cells [49]. Moreover, the control group of A549-implanted mice had more lung metastases than the CDDP therapy group, indicating more aggressive tumors [49]. In mice given CU17 monotherapy, no tissue was injured, while Gem groups showed necrosis, impaired basal membrane, hypertrophy, hematopoiesis, and localized fibrosis in liver, kidneys, and spleen histopathology (Figure 5B–D). Furthermore, Gem alone killed mice, while CU17 or combination treatments did not affect the survival of nude mice (Figure 4B). In a previous study, mice that received Gem treatment displayed abnormal and enlarged kidney cells [50]. These cells exhibited cytoplasmic vacuoles or eosinophilic inclusions, as well as degenerative nuclei with localized karyopyknosis [50]. In addition, the liver and kidney sections of Gem-treated mice exhibited a disruption of the hepatic tissue and the presence of necrotic areas. Renal sections of the Gem treatment group showed significant histological changes, including degenerative and necrotic changes in the epithelial lining cells, as previously described [51,52]. Nevertheless, the combination treatments of Gem have shown promise in reducing negative impacts. For example, LPE has been found to effectively reduce hepatotoxicity caused by Gem treatment [12,53]. In this study, after administering Gem in conjunction with CU17, liver, kidney, and spleen tissues exhibited a more consistent cell structure, and the number of damaged tissues decreased (Figure 5B–D). Thus, the co-administration of CU17 and Gem resulted in decreased tumor growth and decreased toxicity to the liver and kidneys. Our findings illustrate the Gem and CU17 combination treatment’s potential to enhance efficacy and minimize toxicity.

## 5. Conclusions

This study reports a novel therapeutic regimen based on the combination of a CU17 and Gem. Numerous functional assays on NSCLC cells revealed that the combination of CU17 and Gem exhibited synergistic antitumor effects. Based on our findings, CU17 could enhance Gem-induced cellular apoptosis and cell cycle arrest in A549 lung cancer cells. The antitumor effects of Gem were enhanced through co-treatment, which involved an increase in the Bax/Bcl-2 expression ratio. The expression of p21, p53, pERK1/2, and Ac-H3 were also found to be increased in response to co-treatment. Notably, an in vivo assessment using a mouse model xenograft tumor indicated that the co-treatment of Gem with CU17 improved its efficacy. Overall, this study suggests that CU17 can act as an adjunct therapy along with Gem for NSCLC. Additional research on large-scale animal models is necessary to validate this combination regimen as an effective treatment for NSCLC.

## Figures and Tables

**Table 1:** Body weight, % body weight change (%BWC), and relative organ weight of nude mice in the vehicle control and treated groups.

Groups	Initial Body Weight (g)	Final Body Weight (g)	% BWC	Organ Index (g/100 g Body Weight)		
				Liver	Kidney	Spleen
Vehicle	25.75 ± 0.13	28.14 ± 0.78	9.29	7.40 ± 0.65	1.00 ± 0.07	0.65 ± 0.04
Gem	24.52 ± 0.92	25.8 ± 1.61	5.19	7.37 ± 0.40	1.08 ± 0.01	1.32 ± 0.41 ^a^
CU17-15	25.45 ± 0.55	28.43 ± 0.80	11.68	8.00 ± 0.26	1.04 ± 0.05	0.68 ± 0.15
CU17-30	24.96 ± 0.45	28.39 ± 0.67	13.72	7.54 ± 0.43	1.03 ± 0.05	0.66 ± 0.06
Gem+CU17-15	24.45 ± 0.25	26.41 ± 0.41	8.01	7.55 ± 0.18	1.05 ± 0.05	1.46 ± 0.23 ^a^
Gem+CU17-30	25.06 ± 1.02	27.39 ± 0.47	9.30	7.82 ± 0.27	1.06 ± 0.11	1.20 ± 0.13 ^a^

Data were expressed as mean ± SD from mice (Vehicle control, n = 4; Gem, n = 3; and CU17-15, CU17-30, Gem+CU17-5, and Gem+CU17-30, n = 5) treated with Gem: Gemcitabine 50 mg/kg, CU17-15: CU17 15 mg/kg, CU17-30: CU17 30 mg/kg, and combination of Gem and CU17 at the indicated concentrations. ^a^ *p* < 0.05, compared with vehicle control.

## Data Availability

The datasets generated and/or examined in the course of this research are available from the corresponding author upon reasonable request.

## Supplementary Materials

The following supporting information can be downloaded at: https://www.mdpi.com/article/10.3390/pharmaceutics17020158/s1, Figure S1: ^1^H NMR spectrum of CU17; Figure S2: ^13^C NMR spectrum of CU17; Figure S3: IR spectrum of CU17; Figure S4: Mass spectrum of CU17.
