# Supplementary material for: Synergistic Anti-Cancer Activities of Curcumin Derivative CU17 Combined with Gemcitabine Against A549 Non-Small-Cell Lung Cancer Cells
Source: Pharmaceutics. 2025 Jan 24;17(2):158. doi: 10.3390/pharmaceutics17020158 (PMC11858881; doi:10.3390/pharmaceutics17020158)
Supplement: Supplementary file 1 [file pharmaceutics-17-00158-s001.zip › pharmaceutics-3437107-supplementary.pdf]

Supplementary

Synergistic Anti-Cancer Activities of Curcumin Derivative CU17 Combined with Gemcitabine Against A549 Non-Small-Cell Lung Cancer Cells

Narissara Namwan [1], Gulsiri Senawong [1], Chanokbhorn Phaosiri [2], Pakit Kumboonma [3], La-or Somsakeesit [4], Arunta Samankul [1], Chadaporn Leerat [1] and Thanaset Senawong [1,*]

1 Department of Biochemistry, Faculty of Science, Khon Kaen University, Khon Kaen 40002, Thailand; narissaranamwan@kkumail.com (N.N.); gulsiri@kku.ac.th (G.S.); s_arunta@kkumail.com (A.S.); l_chadapornee@kkumail.com (C.L.)
2 Department of Chemistry, Faculty of Science, Khon Kaen University, Khon Kaen 40002, Thailand; chapha@kku.ac.th
3 Department of Applied Chemistry, Faculty of Science and Liberal Arts, Rajamangala University
of Technology Isan, Nakhon Ratchasima 30000, Thailand; pakit.ku@rmuti.ac.th
4 Department of Chemistry, Faculty of Engineering, Rajamangala University of Technology Isan,
Khon Kaen 40000, Thailand; laor.so@rmuti.ac.th
* Correspondence: sthanaset@kku.ac.th

CU17 was synthesized by reacting curcumin with 2-aminothiophenol under a refluxed condition. Purification of the crude product by column chromatography (5% MeOH in CH_2Cl_2) gave **CU17** in 82% yield.

(4*Z*,6*E*)-5-Hydroxy-1,7-bis(4-hydroxy-3-methoxyphenyl)-1-((2-mercaptophenyl)amino)hepta-4,6-dien-3-one (**CU17**) : R_f = 0.50 (5% MeOH in CH_2Cl_2). IR (neat) υ_{max} 3288 (OH), 1738 (C=O), 1560 (Ar), 1508 (Ar), 1265 (C-O) cm^{-1}. ^{1}H NMR ($CDCl_3$, 400 MHz) δ 7.54 (dd, J = 2.0, 8.0 Hz, 1H), 7.39 (m, 2H), 7.14 (m, 3H), 7.01 (dd, J = 2.0, 8.0 Hz, 1H), 6.90 (s, 1H), 6.76 (d, J = 8.0 Hz, 1H), 6.69 (s, 2H), 6.63 (d, J = 12.0 Hz, 1H), 5.48 (s, 1H), 4.71 (dd, J = 4.0, 8.0 Hz, 1H), 3.83 (s, 3H), 3.72 (s, 3H), 2.75 (dd, J = 4.0, 12.0 Hz, 1H), 2.61 (dd, J = 8.0, 12.0 Hz, 1H). ^{13}C NMR ($CDCl_3$, 100 MHz) δ 188.55 (C-3), 161.57 (C-5), 149.78 (C-3″), 148.22 (C-4″), 147.47 (C-3′), 145.96 (C-4′), 141.75 (C-1‴), 139.95 (C-7), 135.02 (C-1″), 134.98 (C-1′), 129.94 (C-3‴), 126.77 (C-6‴), 126.74 (C-5‴), 125.93 (C-6″), 124.43 (C-6′), 123.17 (C-4‴), 122.53 (C-5″), 119.03 (C-

5′), 115.42 (C-2‴), 114.66 (C-2″), 110.26 (C-2′), 110.12 (C-6), 98.00 (C-4), 55.01 (C-1), 54.92 (OCH_3), 40.15 (C-2). HRMS-ESI (m/z) $[M - H_2O + H]^+$ calcd for $C_{27}H_{26}NO_5S$ 476.1531, found 476.1574.

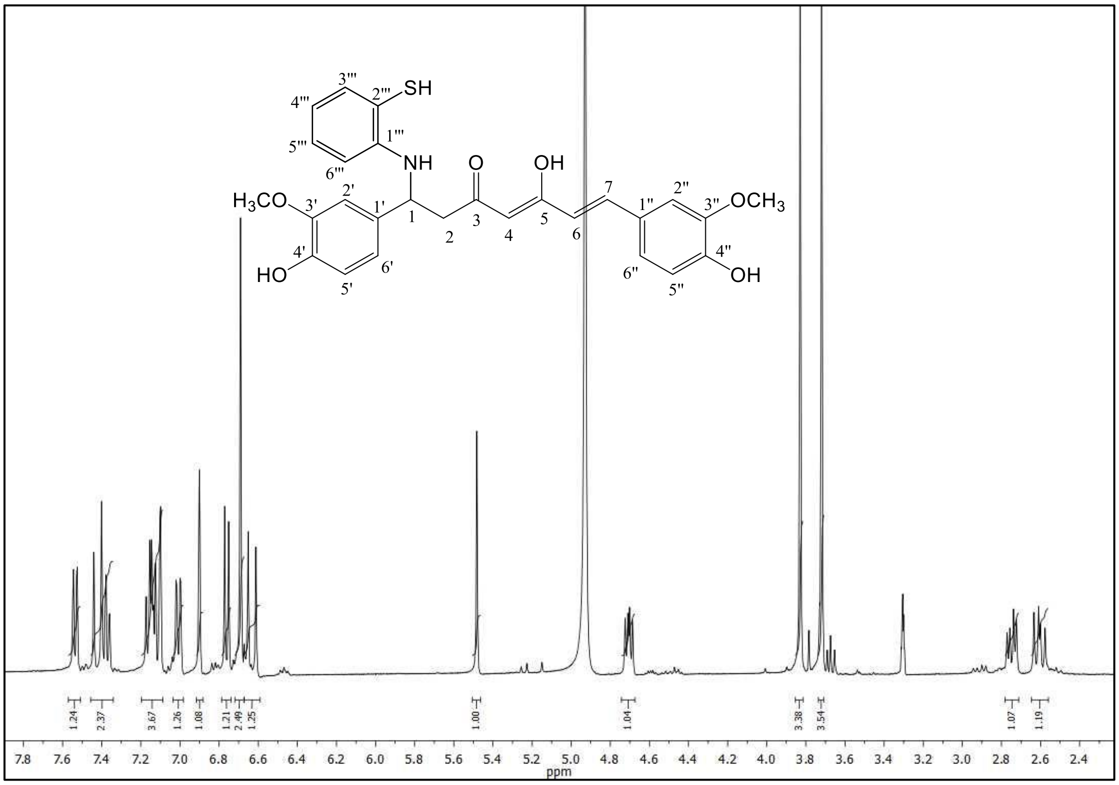

Figure S1. ^{1}H NMR spectrum of CU**17**

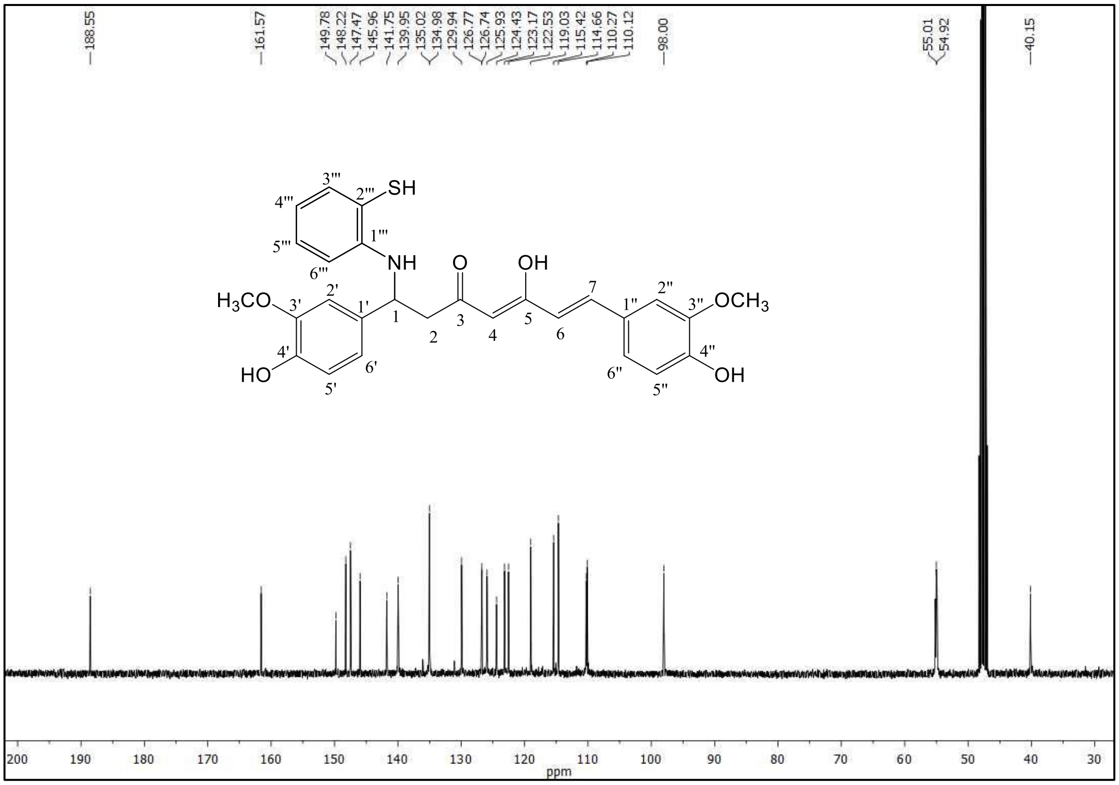

Figure S2. ^{13}C NMR spectrum of CU**17**

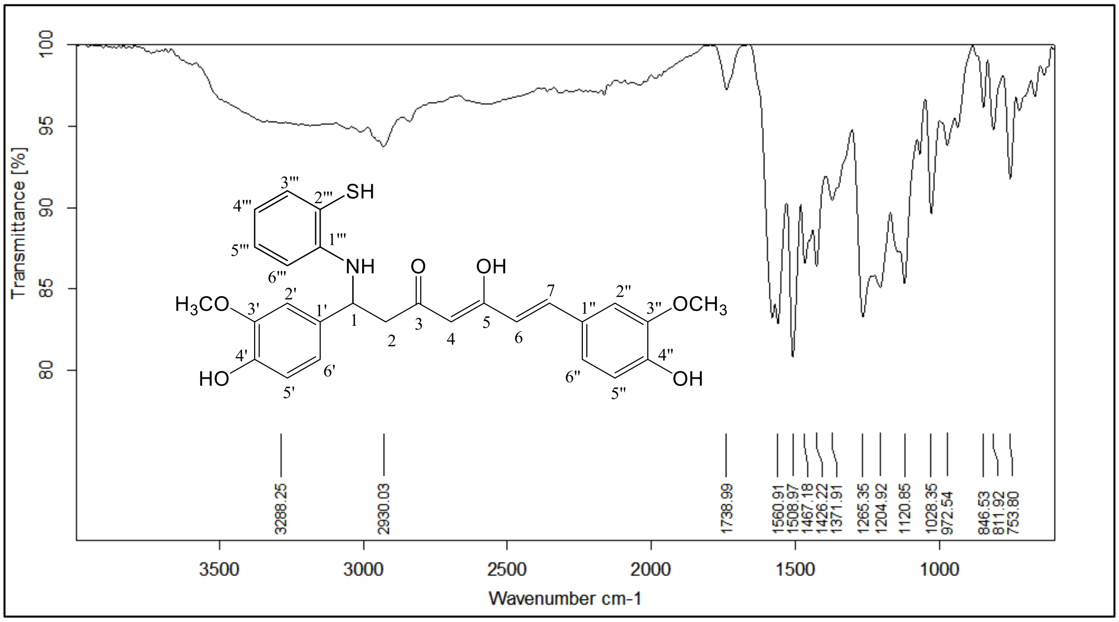

Figure S3. IR spectrum of CU**17**

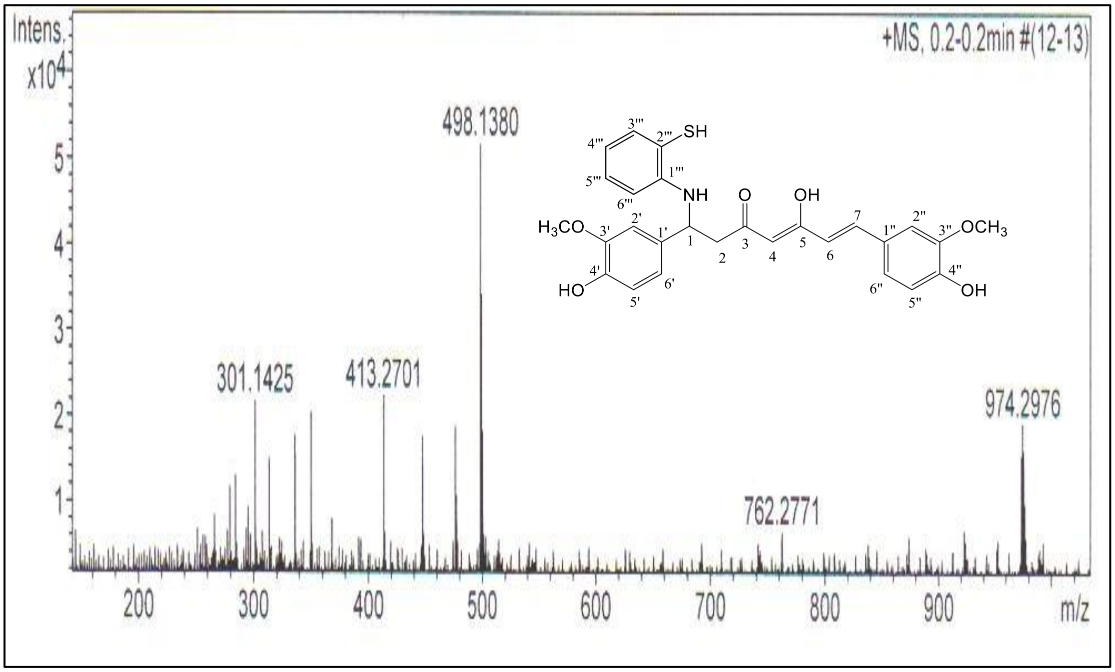

Figure S4. Mass spectrum of CU**17**
